# Hydrolysed Collagen from Sheepskins as a Source of Functional Peptides with Antioxidant Activity

**DOI:** 10.3390/ijms20163931

**Published:** 2019-08-13

**Authors:** Arely León-López, Lucía Fuentes-Jiménez, Alma Delia Hernández-Fuentes, Rafael G. Campos-Montiel, Gabriel Aguirre-Álvarez

**Affiliations:** 1 Universidad Autónoma del Estado de Hidalgo, Instituto de Ciencias Agropecuarias, Av. Universidad Km. 1 Rancho Universitario, Tulancingo, Hidalgo 43600, Mexico; 2 Instituto Tecnológico Superior del Oriente del Estado de Hidalgo, Carretera Apan-Tepeapulco Km 3.5, Las Peñitas, Ápan, Hidalgo 43900, Mexico

**Keywords:** collagen, hydrolysis, enzyme, molecular weight, sheepskin

## Abstract

The extraction and enzymatic hydrolysis of collagen from sheepskins at different times of hydrolysis (0, 10, 15, 20, 30 min, 1, 2, 3 and 4 h) were investigated in terms of amino acid content (hydroxyproline), isoelectric point, molecular weight (Mw) by sodium dodecyl sulphate polyacrylamide gel electrophoresis (SDS-PAGE) method, viscosity, Fourier-transform infrared (FTIR) spectroscopy, antioxidant capacity by 2,2′-azino-bis(3-ethylbenzothiazoline-6-sulphonic acid) (ABTS) and 2,2-diphenyl-1-picrylhydrazyl (DPPH) assays, thermal properties (Differential Scanning Calorimetry) and morphology by scanning electron microscopy (SEM) technique. The kinetics of hydrolysis showed an increase in the protein and hydroxyproline concentration as the hydrolysis time increased to 4 h. FTIR spectra allowed us to identify the functional groups of hydrolysed collagen (HC) in the amide I region for collagen. The isoelectric point shifted to lower values compared to the native collagen precursor. The change in molecular weight and viscosity from time 0 min to 4 h promoted important antioxidant activity in the resulting HC. The lower the Mw, the greater the ability to donate an electron or hydrogen to stabilize radicals. From the SEM images it was evident that HC after 2 h had a porous and spongy structure. These results suggest that HC could be a good alternative to replace HC from typical sources like pigs, cows and fish.

## 1. Introduction

Collagen is the most abundant protein in bones and connective tissue in vertebrates, and there are at least 29 types. They are different in terms of their amino acid sequence and composition, the function in the organism, and the structure [1,2]. The structure of collagen is a triple helix formed for 3 α chains Gly-X-Y, where X is proline, Y is mainly hydroxyproline, and the triple helix is stabilized for hydrogen bonds with continuous repetition of the Gly-X-Y depending on the collagen type [3]. Collagen can be extracted from the skins, bones, tendons and cartilages of pigs [4], cows [5], marine organisms [6,7,8] and rabbits [9]. Hydrolysed collagen (HC) refers to a group of peptides that results from the proteolysis of native collagen type 1; its molecular weight (Mw) varies from 0.3 to 8 KDa [10]. It does not jellify in solution at room temperature and is soluble in cold water, so it can mix easily with other products [11,12,13]. Additionally, hydrolysed collagen has a neutral smell, is colourless, and can be used in emulsions as a stabilizer. It is widely used in the pharmaceutical industry for the treatment of diseases like osteoarthritis and osteoporosis. Also, in the cosmetics and food industries it is applied for the preparation of fruity beverages and nutritional supplements [14,15,16,17].

The antioxidant activity is the capacity of a substance to inhibit oxidative degradation by reacting with free radicals. There are natural antioxidants such as HC that exhibit mechanisms to exert antioxidant activity hydrogen transfer or electron donation [18]. The antioxidant activity of hydrolysed collagen is generally associated with the molecular weight. Peptides with 2 to 10 amino acid residues have a molecular weight of around 10 KDa. They show high radical scavenging because of their accessibility to active radicals. The amino acid content as well as the Mw of HC are properties closely related to the antioxidant activity [19,20]. 

Several studies on antioxidant activity have been conducted with HC from different sources such as pigs [4,21], cows [22], fish [23,24,25], and invertebrates like jellyfishes or sponges [26,27]. However, less is known about the properties of hydrolysed collagen extracted from ovine sources and its possible applications. The objective of this research is the extraction and hydrolysis of collagen from sheepskins to establish the antioxidant activity as well as the physicochemical properties of the obtained peptides as a function of the hydrolysis time. These results could be of interest in developing an alternative source to fish, cows and pigs.

## 2. Results and Discussion

### 2.1. Protein Content

**Figure 1:**
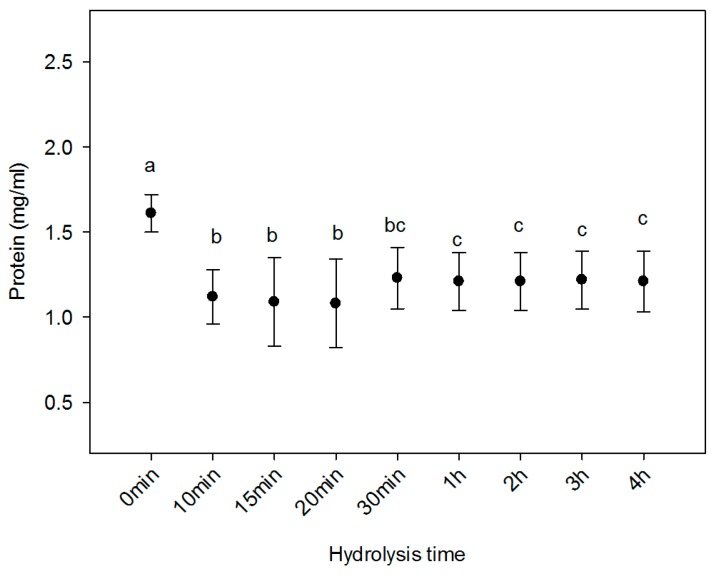
Protein concentration for different hydrolysis times in ovine collagen. Different letters represent the average of three replicates and indicate significant difference at *p* ≤ 0.05.

From Figure 1, it can be seen that the protein concentration of HC was affected by the hydrolysis time. Before hydrolysis (at 0 min), the protein concentration was 1.61 mg/mL. However, in the first 20 min the lowest concentration was reported, up to approximately 1.1 mg/mL. The concentration remained constant around 1.21 mg/mL and there were no statistical differences (*p* > 0.05) from 30 min treatment to the end of the experiment (4 h). This behaviour could be due to the decrease in available substrate and/or enzyme autodigestion [28]. All the treatments in this experiment reported higher protein concentrations compared to those reported by Paul and co-workers [29]. They obtained hydrolysed collagen from cowhide using an enzymatic treatment. The protein concentration was reported with 0.11 mg/mL. Other works [30] also obtained a low protein concentration (around 0.76 mg/mL) from chicken connective tissue with enzymatic treatment at pH 7.5. These results suggest that hydrolysis of collagen from sheepskin under the conditions described above was efficient compared to chicken and bovine sources.

### 2.2. Hydroxyproline Content

**Figure 2:**
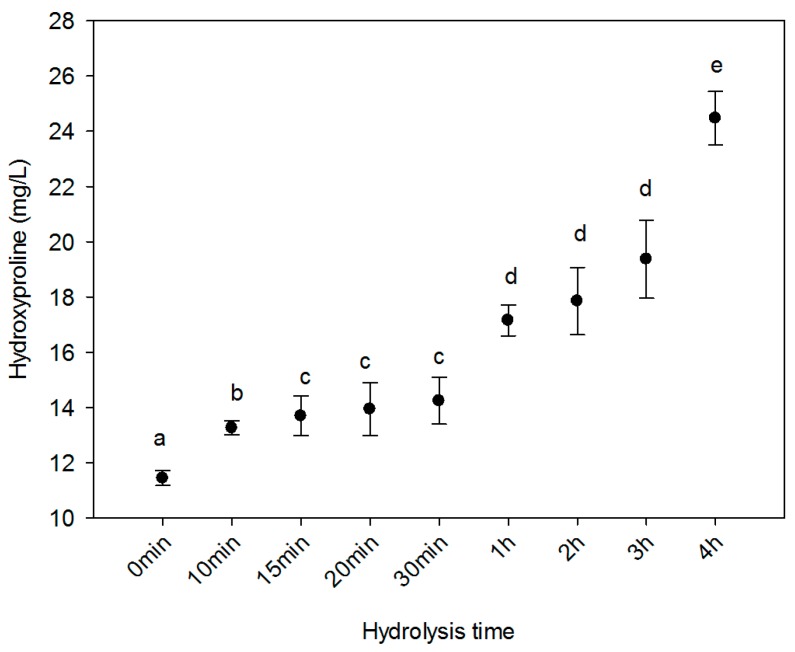
Hydroxyproline concentration in hydrolysed collagen. Different letters indicate significant difference at *p* ≤ 0.05.

Collagen is different from other proteins due to its high concentration of hydroxyproline. This amino acid provides thermal stability to collagen molecules because of the hydrogen bond formation and the presence of an hydroxyl group (OH), limiting the rotation of the peptide chain [15,31]. The hydrolysis of collagen reported in Figure 2 indicates that the longer the hydrolysis time, the more hydroxyproline is obtained. After 4 h of enzymatic and thermal treatment, HC reported 24.47 mg/L. This trend agrees well with previous works carried out on fish bone gelatine [32] and pig collagen [33]. They found a significant increment in hydroxyproline content as the hydrolysis time increased. Also, their maximum yield of this amino acid was found at 4 h of thermal treatment. This increment in hydroxyproline content could be attributed to the hydrolysis of the polypeptide chain. These thermal and enzymatic treatments increased the detectable amount of hydroxyproline. This could be the case with some marine sources like Atlantic salmon skin [34] and bigeye snapper skin [35], for which values of 88.24 mg/mL and 87.75–90.86 mg/mL, respectively, were reported. However, Gómez-Lizárraga and co-workers [36] obtained 1.18 mg/L from bovine tendons.

### 2.3. Amino Acid Content in Hydrolysed Collagen as a Function of Hydrolysis Time

The amino acid composition of hydrolysed collagen (HC) from sheepskin was very similar to other vertebrate collagen sources such as pigs [33], chicken [37], calves [38], cows [39] and fish [40]. Seventeen amino acids were identified and quantified as structural components of ovine collagen. These amino acids were monitored during the enzymatic hydrolysis process of collagen. Results after 1 h were not included in Table 1 because no significant differences (*p* > 0.05) were observed. After 1 m of hydrolysis, serine was detected as the major component (19.33 mg/g of protein). The enzymatic treatment of collagen showed significant increments in aspartic acid and glutamic acid due to enzymatic cleavage of the polypeptide chains of collagen fibres [41]. These amino acids increased their concentrations considerably as a result of the deamidation process of asparagine and glutamine, respectively [42]. Some amino acids such as lysine, proline, cysteine, tyrosine, valine, methionine, isoleucine, leucine and phenylalanine were sensitive to hydrolysis and their concentrations decreased considerably. The same behaviour was observed with HC from fish skin [43].

### 2.4. Isoelectric Point

**Figure 3:**
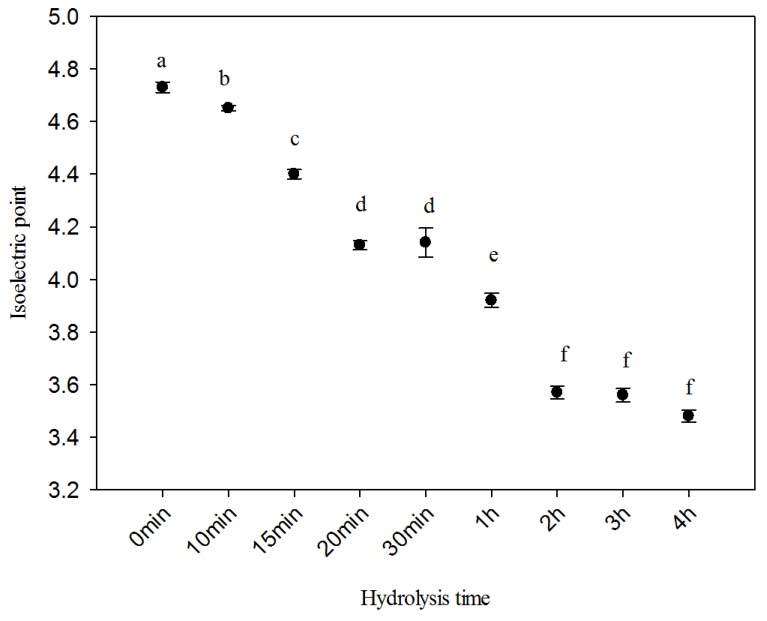
Isoelectric point of ovine collagen hydrolysates as a function of hydrolysis time. Different letters indicate significant difference at *p* ≤ 0.05.

Isoelectric point (pI) is the pH of the collagen molecule at 0 charge. Looking at Figure 3, the pI shifted from 4.61 to 3.68 at the end of the hydrolysis (4 h). Native collagen (0 min) reported a pI value of about 4.7. Similar values of pI for acid (4.9) and pepsin-soluble (5.7) collagen were reported in the literature on the extraction of collagen from ovine bones [1]. Hydrolysed collagen (HC) is an amphoteric macromolecule composed of both acidic (COOH) and basic (NH_3_) functional groups and the pI decrement could be due to the deamination process [10]. When HC was treated at high temperature, the asparagine groups transformed to aspartic acid and the glutamine groups into glutamic acid [42]. This leads to a loss of amino groups and a large relative increase in the carboxyl groups, or a higher content of acidic amino acids, which become dominant, shifting the pI to lower values [44,45]. Collagen is an amphoteric macromolecule that possesses different pI according to the hydrolysis time. The higher the pI, the higher the viscosity observed due to stronger electrostatic repulsions between collagen chains [46].

### 2.5. Molecular Weight and Viscosity

**Figure 4:**
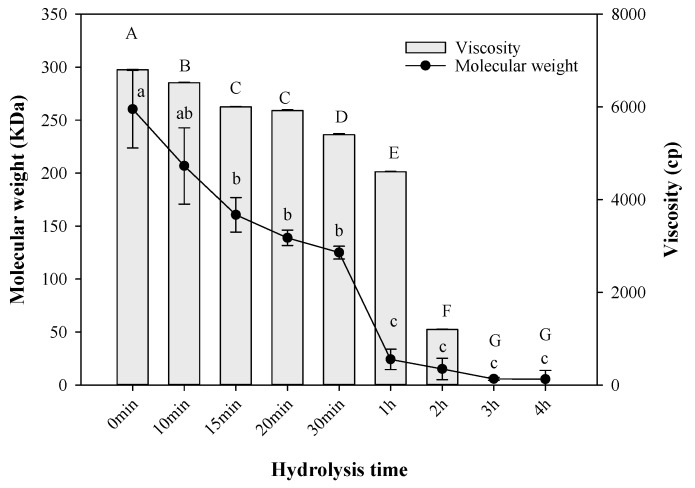
Co-relation between molecular weight and viscosity during the hydrolysis of ovine collagen. The different letters indicate significant difference at *p* ≤ 0.05.

Hydrolysis of collagen is characterized by a reduction in its molecular weight (Mw). Changes in collagen Mw were monitored by SDS-PAGE methodology. Figure 4 shows that native collagen (0 min) reflected the highest Mw with 260.33 KDa. When the hydrolysis started, the Mw dropped to lower values. The first significant changes (*p* ≤ 0.05) were observed at 15 min and 20 min with 160.67 KDa and 138.89 KDa, respectively. However, there was a massive decrement in Mw after treatment for 2 h (15.20 KDa). After this time, no significant changes in Mw were registered (*p* ≥ 0.05) up to 4 h with 5.62 KDa. These results are in good agreement with those reported in the literature with values between 3 and 6 KDa [11,13]. Chi and co-workers [47] used trypsin for digestion to obtain fish hydrolysed collagen with Mw around 14 KDa. Also, hydrolysed collagen from turkey byproducts was obtained with Mw of 34 KDa by using different enzymes [48]. Previous works carried out on Alaska Pollack skin [43] and sea cucumber [49] found high antioxidative properties in HC with Mw around 6‒8 KDa and 5 KDa, respectively. There is a close relationship between the Mw and the viscosity of the collagen [12]. At 0 min, the viscosity reported 6800 Cp. This higher viscosity could be attributed to the presence of high molecular weight and chains [50]. The viscosity decreased upon heating, in accordance with the hydrolysis time. It shifted to 0.5 Cp when the hydrolysis had been going on for 1 h. After this time, no significant changes (*p* ≥ 0.05) were observed. The triple helix structure of native collagen was changed to a random coil form due to the dissociation of the hydrogen bonds [51].

### 2.6. Fourier Transform-Infrared Spectroscopy (FTIR)

**Figure 5:**
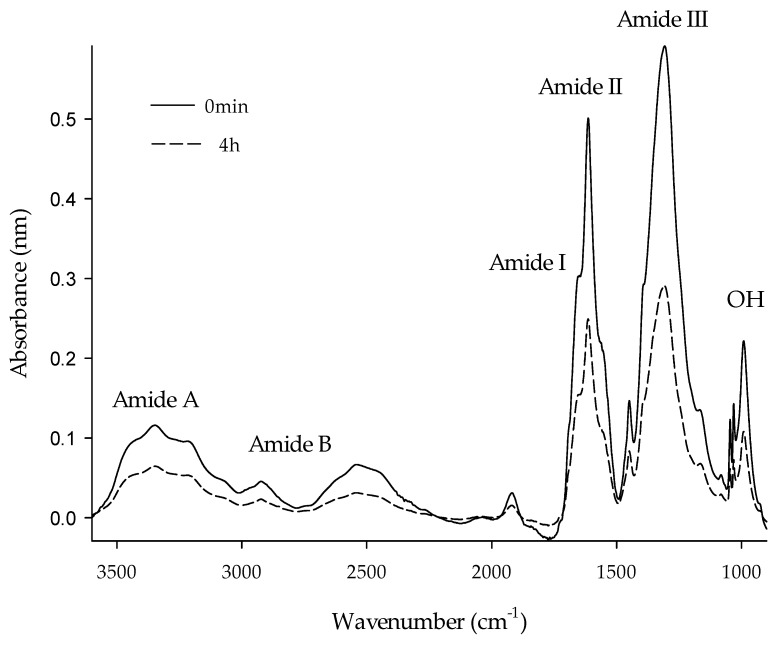
Fourier transform infrared spectra of collagen hydrolysates from 0 min, and 1 h of hydrolysis time.

The FTIR spectra of both native collagen (0 min) and hydrolysed collagen (HC) were produced in the range of 600‒4000 cm^−1^. All FTIR spectra of HC samples overlapped each other. However, for reasons of clarity, Figure 5 only shows the range of 1000‒3500 cm^−1^ for the samples at 0 min and 4 h. There were no changes in peak location for the amide bands between the control and the treated sample. However, the magnitude of amplitude in HC decreased significantly. Amide I at wavelength 1641 cm^−1^ was interpreted as the stretching vibrations of the carbonyl groups (C=O) along the polypeptide backbone. This band is characteristic of α-helix chains and is widely used to analyse the secondary structure of collagen [52]. Amide II was detected at 1548 cm^−1^ for the stretching vibrations of the CN group. Amide III was mainly associated with intermolecular interactions at 1248 cm^−1^, representing the stretching vibrations of the C‒N group and the deformation of the NH group from amide bonds [1]. The amide B (2946 cm^−1^) and Amide A (3295 cm^−1^) bands were related to the asymmetric stretching of the CH_2_ groups and vibrations of tension of the NH group, respectively. The longer the hydrolysis time, the higher the vibrations of OH groups (1037 cm^−1^) reported in the spectra. These results agree very well with the literature [7], suggesting that the HC (1 h) maintained the same characteristics of native collagen (0 min) as the peak locations of all amide bands scarcely caused changes.

### 2.7. Antioxidant Activity

**Figure 6:**
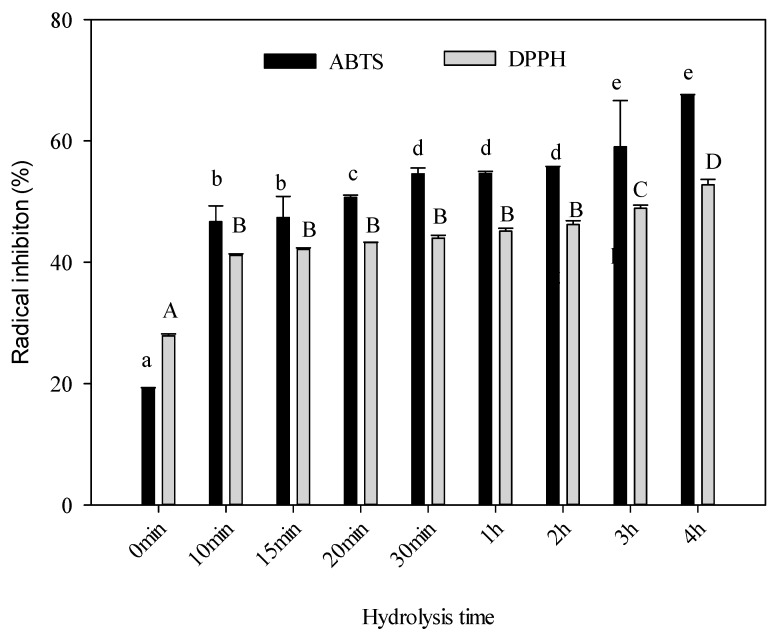
Antioxidant capacity of ovine collagen during hydrolysis. The activity was evaluated with TBS and DPPH methods. Values are expressed as the mean ± SD (*n* = 3). Different letters indicate significant difference at *p* ≤ 0.05.

2,2′-azino-bis(3-ethylbenzothiazoline-6-sulphonic acid) (ABTS) and 2,2-diphenyl-1-picrylhydrazyl (DPPH) assays are methods frequently used in the evaluation of radical scavengers to assess the antioxidant capacity of compounds. The ABTS radical can be applied in a wide range of pH, and is soluble in aqueous and organic media. It allows for the evaluation of both hydrophilic and lipophilic antioxidants [53,54]. DPPH radical is one of the most stable free radicals. It is a simple and quick method that can be used to test the ability of compounds to act as free radical scavengers or hydrogen donors [55]. The antioxidant activity of hydrolysed collagen (HC) was evaluated by the ABTS and DPPH methods as shown in Figure 6. There were differences (*p* < 0.05) between the ABTS and DPPH radical scavenging activities. The highest ABTS and DPPH radical scavenging activity was found at 4 h of hydrolysis with 67.6% and 52.75%, respectively. The ABTS technique reported higher values compared to DPPH. ABTS radical scavenging is commonly used to evaluate the ability of antioxidants to donate an electron or hydrogen atom to stabilize radicals [56]. The antioxidant activity of protein hydrolysates seemed to be affected by the amino acid composition as well as the degree of hydrolysis [33]. The longer the time, the higher the antioxidative activity was observed. It is well known that several amino acids like tyrosine, histidine [57] and lysine possess antioxidant properties [43]. Also, some hydrophobic amino acids like isoleucine and methionine could donate electrons or hydrogen, converting the radical to a more stable species and contributing to higher radical scavenging [58]. The amino acid content results of this research showed that strong hydrolysis (4 h) of collagen from sheepskin increased the concentration of these amino acids. At this time (4 h), the radical scavenging activity increased significantly because there was an increment of glutamic acid from 7.99 to 16.58 mg/g of protein. On the other hand, the enzymatic treatment of native collagen decreased its Mw by around 6 KDa. Previous works carried out with different sources such as fish [6,8] and squid [59] showed that Mw was one of the most important parameters that determined the biological activity of collagen [60]. The lower the Mw polypeptides, the higher the antioxidant activity was found to be. These results suggested that hydrolysis of collagen generated a wide variety of smaller peptides and free amino acids depending on the hydrolysis time [61]. Therefore, the composition of amino acid content, degree of hydrolysis and size of collagen chains and source of raw material could define the antioxidant capacity of the HC.

### 2.8. Thermal Properties

The thermal properties of hydrolysed collagen was evaluated in dried powder with 0% of water content (d.b.) from samples obtained after different hydrolysis times. From Table 2, it can be seen that the melting temperature (Tm) reduced gradually from 153.3 °C (0 min) to 136.9 °C as the hydrolysis time increased up to 4 h. This reduction in Tm suggested that the thermal stability of the triple helical structure of collagen was affected after 1 h of thermal and enzymatic treatment (137.2 °C). It is well known that intermolecular helix formation is dependent on the molecular weight (Mw) of alpha chains [62]. The higher the Mw, the higher the Tm values that will be observed. The Tm results suggested that intermolecular helix formation reduced significantly as hydrolysis took place. This reduction originated at the bimolecular nucleation stage, which involved an intramolecular β-turn facilitated by glycine and/or proline residues [63]. This means that propagation was far less effective compared with native collagen at 0 min of treatment. The DSC thermograms of this native collagen showed a narrow melting range, suggesting a more homogenous population of longer helical segments. However, as the hydrolysis process started, the samples showed a broad melting range, indicating a wide molecular weight distribution of helix lengths by shifting the Tm towards lower values.

Considering the enthalpy as the energy required to disorganize the helical structure, it was possible to assume that native collagen possessed a more ordered structure (20.06 J/g). However, hydrolysis treatment produced low molecular weight residues with a low possibility of intramolecular refolding. In fact, previous work [63] has suggested a limit of 40‒80 amino acid residues as the critical size of nuclei for renaturation. Also, we have seen that samples with Mw < 15 KDa cannot recover their helical conformation even at high concentrations [64]. Enthalpy of samples with 4 h of hydrolysis showed significant differences (*p* < 0.05) with the lowest degree of reorganization (8.93 J/g). However, it still showed some energy requirements to disorganize its structure. This structural conformation could be due to the intermolecular interaction of two or three strands with low Mw [64].

### 2.9. SEM Images

**Figure 7:**
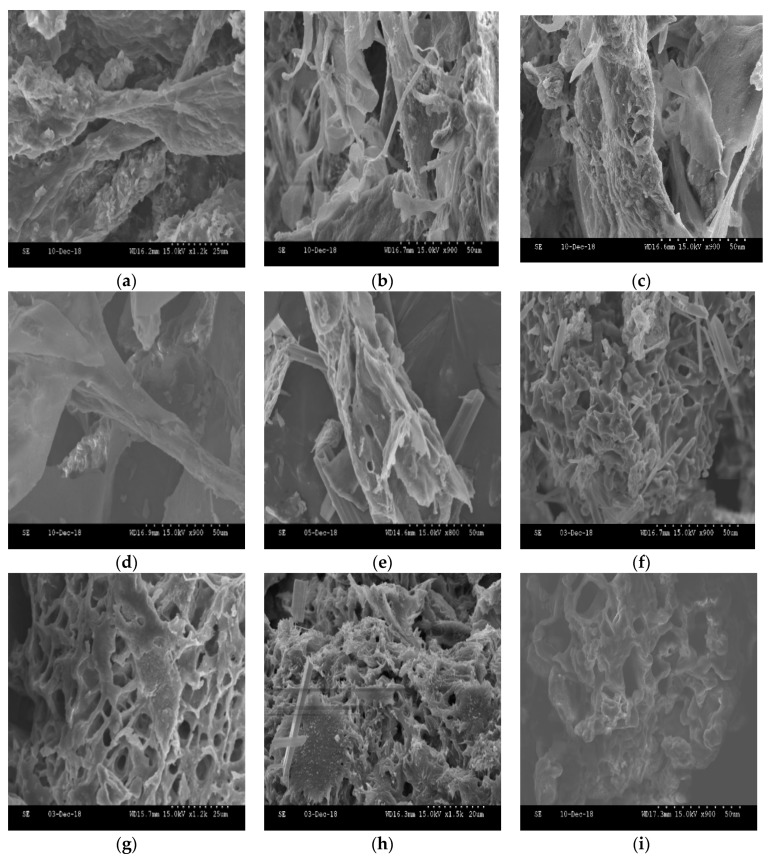
HC morphology changes during the hydrolysis alkaline treatment: (**a**) 0 min, (**b**) 10 min, (**c**) 15 min, (**d**) 20 min, (**e**) 30 min, (**f**) 1 h, (**g**) 2 h, (**h**) 3 h, (**i**) 4 h.

The morphological appearance of native collagen and their resulting hydrolysates are shown in Figure 7. It can be seen that HC showed changes in morphology across the different hydrolysis times. During the first 20 min of hydrolysis, the collagen did not appear to have pores in its structure (Figure 7a‒c). However, after 30 min of treatment (Figure 7d‒f), initial degradation of collagen was seen in the form of small pores in the protein structure, the result of enzymatic action leading to a partial disassembly of fibres into fibrils, and therefore, the generation of low molecular weight polypeptides.

After 2 h of enzymatic hydrolysis (Figure 7h,i), the disaggregation of collagen fibrils within the collagen fibres was evidence of the autolysis of the enzymatic treatment [7]. The collagen structure of these treatments (after 3 h and 4 h) appeared to be extremely degraded, with a spongy and porous form. Previous works carried out with marine sources [65] discovered that the Mw of peptides influences the properties of HC. The lower the Mw, the more pores there are, and the more open the structure observed in the images.

### 2.10. Relationship between Mw, Viscosity, Antioxidant Activity and Thermal Properties

The properties of HC mainly appeared to obey the Mw of polypeptide chains obtained after hydrolysis. However, a strong relationship was observed with the other parameters evaluated; viscosity was closely related to Mw because these results suggested that the low Mw of polypeptide chains produced a low hydrodynamic volume of collagen molecules in solution [42]. This could be why the viscosity dropped to close to 0 Cp after 1 h of hydrolysis. The antioxidant activity also appeared to be dependent on the Mw parameter. The results from this research showed that the lower the Mw, the higher the antioxidant activity of HC. This behaviour was confirmed by the higher concentrations of hydroxyproline after 1 h of hydrolysis. Additionally, higher concentrations of aspartic acid and glutamic acid were obtained over the same period of time. The measurement of thermal properties in HC indicated that low-Mw samples (2 h, 3 h and 4 h) resulted in the lowest enthalpy. This means that less energy was required to disorganize the structure of HC because its low Mw avoided the organization into a triple helical form [66].

## 3. Materials and Methods

Sheepskins with 40–50% water content were used in this experiment. They were obtained as byproducts from a local market in Tulancingo, Hidalgo, Mexico. Reactive grade acetic acid (99% purity), sodium chloride reactive grade, porcine digestive protease (pepsin), dialysis membrane tubing with 6–8 kDa molecular weight cut-off, 4-dimethylaminobenzaldehyde at 5%, 65%, perchloric acid, 0.006 M chloramine T, 0.8 M and citrate buffer were purchased from Sigma-Aldrich Corp. (MA, USA).

### 3.1. Conditioning of The Skin before Collagen Extraction 

The sheepskins were soaked to recover up to 70% water, followed by a fleshing process to remove the connective tissue and fat. Then, the skins were shaved to remove most of the hair.

### 3.2. Collagen Extraction from Ovine Skin

The methodology of Chuaychan et al. [67] was used, with some modifications. Pre-treated sheepskin was cut into small squares of approximately 1 cm^2^ and suspended in 0.5 M acetic acid solution at a ratio of 1:10 (*w/v*). The sample was placed in a shaker machine (Bellco Biotechnology. NJ, USA) at 140 rpm for 3 h at room temperature (20 ± 2 °C), followed by the addition of pepsin at a concentration of 1 g/L with gentle stirring for 48 h. The sample was filtered and precipitated with a solution of 2.6 M sodium chloride. The precipitated material was centrifuged for 15 min at a relative centrifugal force of 3380× *g* in a centrifuge model Z36HK (HERMLE Labortechnik GmbH, Wehingen, Germany).

### 3.3. Hydrolysis of Collagen

Precipitated collagen was re-suspended in a solution of 1 M NaCO_3_ at a ratio of 1:4 (*w/v*). The pH was adjusted to 8 ± 0.2. The hydrolysis of collagen was carried out with the enzyme trypsin at a concentration of 1:50 (*w/v*) in a water bath at 60 °C for different times, as follows: 10 min, 15 min, 20 min, 30 min, 1 h, 2 h, 3 h and 4 h. The control sample was called 0 min. All the samples were inactivated at 90 °C for 10 min and stored at 4 °C.

### 3.4. Protein Determination

Following Bradford determination [68], 5 mL of Bradford reagent and 100 µL of the sample were added and mixed in a vortex for 2 min. After 5 min storage in darkness, the sample was read in a spectrophotometer at 595 nm. The serum albumin at different concentrations was used to create a calibration curve.

### 3.5. Isoelectric Point 

The isoelectric point was measured by using a Zetasizer nano ZS90 coupled to auto-titrator MPT-2. Laser Doppler and a DTS1070 cell (Worcestershire, UK) were used to determinate the electrophoretic mobility. HC was diluted in distilled water at 1:10. Different values of pH from 2 to 7 were obtained by using 0.5 M HCl and 0.75 M NaOH buffers, respectively.

### 3.6. Hydroxyproline Quantification

According to the AOAC methodology [69], 4 g of the sample and 30 mL of 3.5M sulphuric acid were placed in an oven at 105 °C for 12 h. The volume was adjusted to 500 mL with distilled water and filtered. Two millilitres of filtered sample were mixed with 1 mL of oxidant solution (0.006 M chloramine T in 0.8 M citrate buffer, pH 6.0) in a reaction tube. The volume was adjusted to 100 mL and stirred for 30 min at room temperature. Two millilitres of colour reagent (10 g of dimethylamine benzaldehyde in 35 mL of 65% perchloric acid) were added with stirring at 60 °C for 15 min. The absorbance of samples was measured against the blank at 558 nm in a UV-visible Jenway Genova, (Bibby Scientific, Staffordshire UK) spectrophotometer.

The collagen content was calculated with the next equation:

(1) $$\%\;Hydroxyproline=\frac{\left(Y\right)\left(2.5\right)}{\left(W\right)\left(V\right)},$$

where:

*Y* = Hydroxyproline concentration from the standard curve
*W* = sample weight
*V* = volume in mL to adjust the 100 mL

### 3.7. Viscosity Analysis 

Viscosity measurement was carried out using a viscometer Brookfield RTV (MA, USA) (spindle number: 5; speed of 100 rpm). Viscosity was expressed in centipoise (cP). All the samples were previously conditioned at 7 °C [70].

### 3.8. Molecular Weight

The sodium dodecyl sulphate polyacrylamide gel electrophoresis (SDS-PAGE) determination was performed according to Laemmli methodology [71]. One millilitre of dialyzed collagen was dissolved in 0.5M Tris–HCl buffer pH 6.8 (1% SDS, 10% glycerol and 0.01% bromophenol blue) and boiled for 5 min. Then, 10 μL of the denatured sample and 5 μL of a marker with a molecular weight from 10 kDa to 220 kDa (BenchMark Protein Ladder, Thermo Scientific, Pierce™, MA, USA) were loaded into wells at the top of the polyacrylamide gel. This gel contained a 4% stacking gel on top of the 12.5% resolving gel. A voltage of 50 V was applied for 30 min, and once the mobility of the proteins reached the resolving layer, the voltage was increased to 100 V for 4 h in order to see the separation of proteins according to size. As the electric current ran through the buffer, the negatively charged proteins migrated towards the anode and lower molecular weight proteins reached the bottom of the gel. After the migration of proteins, the gel was stained with Silver Stain Kit (Thermo Scientific, Pierce™ MA, USA).

### 3.9. Fourier Transform-Infrared (FTIR) Spectroscopy

The FTIR technique offers a green alternative because it allows us to quantify substances without organic solvents. The samples do not require any pretreatment, thus reducing the environmental damage caused by toxic waste. Also, it is a fast technique based on the natural vibrational frequencies of the chemical bonds present in molecules. FTIR is a non-destructive technique using a minimum amount of sample [72]. The absorption spectra by the FTIR technique were obtained with the Frontier FT-MIR (Perkin Elmer. MA, USA) equipment. The wavelength ranged from 380 to 4000 cm^−1^ at room temperature. The samples were brought into intimate contact with the diamond crystal by applying a loading pressure. For each sample, the spectrum represented an average of four scans with 4 cm^−1^ resolution. A spectrum of the empty cell was used as the background. Automatic signals were collected in 3620 scans at 1 cm^−1^ resolution. All the data were processed with Spectrum^TM^ 10 (Perkin Elmer. MA, USA) software.

### 3.10. Differential Scanning Calorimetry (DSC)

Thermal properties of HC were obtained with DSC series Q 2000 with intracooler RCS90 (DE, USA). It was calibrated with indium (Tm, onset ¼ 156.6 8C, ΔH ¼ 28.45 J/g). An average of 1.5 ± 0.1 mg of sample with known water content (0% db) was packed and hermetically sealed in a 50-mL stainless steel pan. An empty, hermetically sealed pan was used for a reference. Both heating and cooling scan rates were performed at 10 °C/min. Two heating scans were performed from 25 °C to 120 °C. Melting point temperature (Tm) and enthalpy (ΔH) were determined with TA 2000 analysis software (TA Instruments, DE, USA) based on the endothermic changes registered in the thermogram. 

### 3.11. Amino Acid Determination

Amino acid content determination was performed according to Cohen [73] with some modifications. Three milligrams of freeze-dried HC were suspended in 6 M HCl and 1% *v/v* phenol at 150 °C for 1 h. Hydrolysed samples were dissolved in 2 mL of 0.5 M citrate buffer. Amino acid content was determined by high-performance liquid chromatography (HPLC) in a Hewlett Packard model GmbH (Winchester, UK) connected to a fluorescence detector (Ex. 250 Em. 395). The derivation reaction was carried out with 20 µL of the sample diluted in 60 µL buffer (borate buffer, Waters, Thermo Scientific, Pierce™, MA, USA) and 1 min stirring. Twenty microlitres of reagent AQC (Waters) were added with stirring for another 1 min, followed by the heating of the sample at 50 °C for 10 min. The amino acid separation was carried out in a Bluespher® column (100 × 2 mm ID) in reverse phase C18 octa-decyl dimethylsilane (Berlin, Germany). Conditions of work: mobile phase A: 50 mM sodium acetate, pH 5.75 and mobile phase B: 50 mM sodium acetate, pH 6/CAN 30:70 *v/v*, and 1 mL/min flow.

### 3.12. Antioxidant Activity

A solution of 2,2′-azino-bis(3-ethylbenzothiazoline-6-sulphonic acid) (ABTS) radical was prepared according to the literature [74] by mixing 7 mM ABTS and 2.45 mM potassium persulfate. After 16 h of stirring at room temperature in the dark, the ABTS solution was diluted with ethanol to stabilize it to 0.70 ± 0.02 at 734 nm. One millilitre of stabilized ABTS solution was mixed with 0.2 mL of the sample and the absorbance raised to 734 nm in a UV-visible Jenway Genova (Bibby Scientific, Staffordshire UK) spectrophotometer. 

For assessing the antioxidant activity by 2,2-diphenyl-1-picrylhydrazyl (DPPH) radical inhibition [75], 0.5 mL of the sample was mixed with 2.5 mL of 6.1 × 10^−5^ M methanolic radical DPPH solution and maintained in darkness for 30 min. The absorbance was measured at 515 nm in a UV-visible Jenway Genova spectrophotometer. The antioxidant activity for ABTS and DPPH radical inhibition was calculated via the following equation:

(2) $$\%\;\mathrm{Inhibition}=\frac{\mathrm{Initial}\;\mathrm{absorbance}-\mathrm{Final}\;\mathrm{absorbance}}{\mathrm{Initial}\;\mathrm{absorbance}}\times 100$$

### 3.13. Scanning Electron Microscopy (SEM) 

Morphology analysis was observed by a scanning electron microscope (Model S-2600N, HITACHI, Tokio, Japan). Freeze-dried HC samples were mounted on a strip of self-adhesive carbon paper and sputter-coated with gold to be observed in the scanning electron microscope at an acceleration voltage of 15 kV.

### 3.14. Statistical Analysis

A randomized design experiment and an analysis of variance (ANOVA) were applied to the experimental data, which included a Tukey test (*p* ≤ 0.05). Data were analysed with SPSS 16.0 software (SPSS Inc., Chicago, IL, USA). Three replicates per treatment were considered in this experiment.

## 4. Conclusions

The study demonstrated that sheepskins are a good source of hydrolysed collagen. The best results were seen after 2 h of hydrolysis treatment. From this point, the kinetics of native collagen hydrolysis produced polypeptides with a low molecular weight and viscosity. This reduction in the polypeptide chain size affected the thermal properties of HC as the 4 h treatment produced a lower enthalpy value. Also, the antioxidant properties of HC were enhanced as the hydrolysis time increased. The functional properties of HC could be controlled by the hydrolysis time and sheepskins appeared to be a good alternative to typical sources like pigs, cows and fish.

## Figures and Tables

**Table 1:** Amino acid content (mg of amino acid/g of protein) of hydrolysed collagen as a function of hydrolysis time.

Amino Acid	0 min	10 min	20 min	30 min	1 h
Aspartic acid	4.04 ± 0.03 ^a^	10.86 ± 0.05 ^d^	5.06 ± 0.04 ^f^	28.03 ± 0.036 ^g^	32.85 ± 0.16 ^a^
Glutamic acid	7.99 ± 0.06 ^a^	7.71 ± 0.04 ^a^	16.19 ± 0.05 ^e^	38.21 ± 0.048 ^f^	16.58 ± 0.03 ^a^
Serine	19.33 ± 0.16 ^a^	25.99 ± 0.03 ^c^	17.45 ± 0.03 ^a^	13.72 ± 0.03 ^a^	17.87 ± 0.05 ^a^
Glycine	4.86 ± 0.01 ^a^	4.31 ± 0.04 ^a^	16.71 ± 0.04 ^a^	1.92 ± 0.00 ^a^	3.10 ± 0.00 ^a^
Lysine	6.60 ± 0.04 ^a^	5.72 ± 0.02 ^a^	3.32 ± 0.06 ^a^	2.05 ± 0.00 ^a^	1.92 ± 0.00 ^a^
Histidine	3.38 ± 0.03 ^a^	3.25 ± 0.05 ^a^	2.41 ± 0.01 ^a^	3.95 ± 0.02 ^a^	1.59 ± 0.00 ^a^
Threonine	2.72 ± 0.06 ^a^	4.50 ± 0.02 ^a^	2.90 ± 0.01 ^a^	3.18 ± 0.01 ^a^	3.12 ± 0.00 ^a^
Arginine	8.91 ± 0.01 ^a^	3.46 ± 0.05 ^a^	18.62 ± 0.03 ^b^	2.68 ± 0.02 ^a^	11.02 ± 0.02 ^a^
Alanine	1.39 ± 0.04 ^a^	0.40 ± 0.04 ^a^	1.26 ± 0.00 ^a^	0.14 ± 0.00 ^a^	1.39 ± 0.00 ^a^
Proline	2.68 ± 0.04 ^a^	1.23 ± 0.03 ^a^	0.86 ± 0.00 ^a^	0.13 ± 0.00 ^a^	0.49 ± 0.00 ^a^
Cysteine	1.53 ± 0.02 ^a^	1.84 ± 0.03 ^a^	1.00 ± 0.00 ^a^	0.13 ± 0.00 ^a^	0.36 ± 0.00 ^a^
Tyrosine	12.36 ± 0.18 ^a^	3.02 ± 0.02 ^d^	7.24 ± 0.05 ^f^	0.78 ± 0.00 ^g^	2.9 ± 0.01 ^a^
Valine	3.17 ± 0.05 ^a^	2.73 ± 0.01 ^a^	1.67 ± 0.00 ^a^	1.53 ± 0.00 ^a^	1.26 ± 0.01 ^a^
Methionine	7.80 ± 0.04 ^a^	7.48 ± 0.04 ^a^	0.75 ± 0.00 ^c^	1.36 ± 0.00 ^d^	1.65 ± 0.00 ^a^
Isoleucine	6.13 ± 0.03 ^a^	6.49 ± 0.05 ^a^	0.52 ± 0.00 ^b^	0.27 ± 0.00 ^c^	1.21 ± 0.00 ^a^
Leucine	3.41 ± 0.01 ^a^	6.35 ± 0.03 ^b^	1.00 ± 0.00 ^d^	0.24 ± 0.00 ^e^	1.30 ± 0.00 ^a^
Phenylalanine	2.66 ± 0.07 ^a^	3.31 ± 0.04 ^a^	1.66 ± 0.00 ^a^	0.87 ± 0.00 ^a^	0.69 ± 0.00 ^a^

Results are mean values of three replicates’ SD. Values followed by different letters are significantly different according to Tukey’s test (*p* ≤ 0.05).

**Table 2:** Thermal properties of dried hydrolysed collagen powder at different times of hydrolysis. Average value of three replicates. Values followed by different letters are significantly different according to Tukey’s test (*p* ≤ 0.05).

Hydrolysis Time	Tm (°C)	ΔH (J/g)
0 min	153.38 ± 0.40 ^a^	20.06 ± 0.10 ^a^
15 min	147.97 ± 0.80 ^a^	17.21 ± 0.47^ab^
20 min	147.72 ± 0.45 ^a^	17.48 ± 0.18 ^ab^
30 min	147.59 ± 0.78 ^a^	16.91 ± 0.32 ^ab^
1 h	137.91 ± 0.35 ^b^	15.61 ± 0.21 ^ab^
2 h	137.77 ± 0.13 ^b^	11.46 ± 0.49 ^ab^
3 h	137.24 ± 0.37 ^b^	9.09 ± 0.20 ^ab^
4 h	136.91 ± 0.55 ^b^	8.93 ± 0.11 ^b^

## Abbreviations

HC	Hydrolysed collagen
SDS-PAGE	Sodium dodecyl sulphate polyacrylamide gel Electrophoresis
Mw	Molecular weight
FTIR	Fourier transform-infrared spectroscopy
DSC	Differential scanning calorimetry
DPPH	2,2-diphenyl-1-picrylhydrazyl
ABTS	2,2′-azino-bis, 3-ethylbenzothiazoline-6-sulphonic acid
ANOVA	Analysis of variance
SEM	Scanning electron microscopy
